# *Puccinellia tenuiflora* as a Pioneer Grass Species for Saline–Alkali Land Restoration: Adaptive Mechanisms and Post-Restoration Forage Utilization Potential

**DOI:** 10.3390/plants15162447

**Published:** 2026-08-12

**Authors:** Jiayi Chen, Hongxia Zheng, Zhen Qu, Meihong Sun, Xiaofeng Xu

**Affiliations:** 1 Development Center of Plant Germplasm Resources, College of Life Sciences, Shanghai Normal University, Shanghai 200234, China; 15706243896@163.com (J.C.); zhenghongxia2020@foxmail.com (H.Z.); 2 Shigatse Grassland Workstation, Sangzhuzi District, Shigatse 857000, China; cyzquzhen@126.com

**Keywords:** *Puccinellia tenuiflora*, saline–alkali land restoration, pioneer grass species, saline–alkali adaptation, ion homeostasis, organic-acid metabolism, community establishment, forage utilization potential

## Abstract

*Puccinellia tenuiflora* is a perennial halophytic grass commonly regarded as a pioneer species for the ecological restoration of saline–alkali land. Its adaptive capacity and subsequent utilization value are shaped by interacting structural, physiological, molecular, ecological, and management-related factors. This review summarizes recent studies on saline–alkali tolerance in *P. tenuiflora*, with emphasis on root structural barriers, Na^+^/K^+^ homeostasis, osmotic adjustment, organic-acid metabolism, antioxidant defense, ion transport, and multi-omics regulation. To better understand the integrated stress response, we propose a functional framework that distinguishes first-line defenses from downstream cellular repair mechanisms. First-line defenses include root apoplastic barriers (Casparian strips and suberization) that restrict Na^+^ entry, plasma-membrane Na^+^/H^+^ antiporters (e.g., SOS1) that mediate active Na^+^ exclusion, and K^+^-retention mechanisms (e.g., AKT1, HKT2;1) that preserve cytosolic K^+^/Na^+^ homeostasis—these operate rapidly to prevent ion imbalance at the onset of stress. Downstream repair and acclimation mechanisms include osmotic adjustment via compatible solutes (e.g., proline, glycine betaine), organic-acid accumulation (especially citric acid) for pH regulation and chelation, ROS scavenging systems, and proteomic/phosphoproteomic reprogramming that repair stress-induced damage and restore metabolic balance. Furthermore, saline–alkali stress involves both short-term osmotic shock and long-term ionic toxicity, and available evidence suggests a temporal shift in the relative importance of these mechanisms: osmotic adjustment and rapid ion exclusion dominate during the initial hours to days of stress, whereas organic-acid metabolism, ROS buffering, and molecular reprogramming become increasingly important during prolonged exposure, sustaining tissue integrity and enabling long-term persistence. Current evidence indicates that saline–alkali tolerance in *P. tenuiflora* results from the combined action of several processes, including restricted Na^+^ entry, K^+^ retention, organic-acid accumulation, reactive oxygen species homeostasis, and organ-specific molecular responses. This review also discusses the significance of *P. tenuiflora* in community establishment, saline–alkali land restoration, and post-restoration forage utilization. Field studies and limited feeding trials suggest that *P. tenuiflora* can provide biomass and utilization potential after community stabilization. However, based on current evidence, it is more appropriate to define its forage value as a post-restoration utilization extension rather than as that of a fully developed specialized forage crop. Further studies are required on nutritional quality, mineral-element safety, long-term field management, and animal feeding validation.

## 1. Introduction

Salinization and alkalization are common in arid/semi-arid regions and severely constrain agriculture, grassland, and ecological restoration [1,2,3,4,5]. Such sites often combine high salinity with high pH, drought, poor structure, nutrient deficit, and seasonal cold, making it hard for conventional crops/forages to establish stable stands, thus limiting marginal land use [6,7,8,9]. Native salt-tolerant plants that can survive, propagate, and maintain communities in degraded saline–alkali habitats are therefore of considerable value [6,7,8,9]. *P. tenuiflora* can be considered within this group of species.

*P. tenuiflora* is a perennial halophytic grass belonging to the genus *Puccinellia* in the family *Poaceae*, and is a representative native species in saline meadows and alkaline grasslands of northern China. As a pioneer halophyte, it thrives under extreme salt–alkali stress (pH 8.5–10.0, salinity > 1.5%), making it a prime candidate for rehabilitating severely degraded saline lands in the region. Its dense fibrous root system reduces surface salinity, enriches soil organic matter, and stabilizes eroded soils, effectively acting as a “bio-desalinator” that facilitates the establishment of subsequent plant communities. Moreover, beyond its ecological role, it offers high-quality forage (12–18% crude protein) for livestock and serves as a valuable genetic reservoir of salt-tolerance genes for breeding stress-resilient crops [10]. *P. tenuiflora* thrives in saline–alkali soils where common forages fail, due to its fibrous roots, strong tillering, high leaf ratio, and fast regrowth [11,12,13,14]. These traits are not complex, but they are important because they help the species sustain growth under stress and produce a certain amount of aboveground biomass.

The distribution of *P. tenuiflora* is not restricted to local saline–alkali grasslands in China. Available taxonomic and ecological information generally treats this species as a representative halophytic grass of temperate to cold-temperate inland saline–alkali habitats across Eurasia. Its distribution is closely associated with saline meadows, salt marshes, and alkaline lowlands in Northeast China, North China, and Northwest China, and it may extend to inland saline–alkali regions of Mongolia, the Russian Far East, and Siberia [10,11]. This distribution pattern suggests that *P. tenuiflora* is adapted to temperate continental saline–alkali environments rather than to a highly localized habitat. Therefore, studies of its saline–alkali tolerance mechanisms, community establishment, and post-restoration utilization potential can support saline–alkali land management in northern China and may also provide references for the restoration of temperate saline grasslands and the use of halophytic forage resources worldwide [7,8,10,11,12,13,14].

From an applied perspective, field and comparative studies have provided preliminary evaluations of the restoration function and utilization prospects of *P. tenuiflora* [10,11]. Under suitable management, the species can establish relatively stable communities on saline–alkali land [12,13]. Compared with other salt-tolerant grasses, it also shows certain advantages in salt tolerance, yield, and some forage-related traits [14]. However, these results first indicate that *P. tenuiflora* is suitable as pioneer grass for restoration; they do not directly prove that it already has the industrial evaluation basis of a mature specialized forage crop [15,16,17,18,19]. Fresh *P. tenuiflora* increases dry matter, metabolizable energy, and crude protein intake, per feeding trials [20]. Thus, *P. tenuiflora* may be converted into a forage resource after restoration, but its forage value is better understood as a functional extension following ecological restoration.

**Figure 1:**
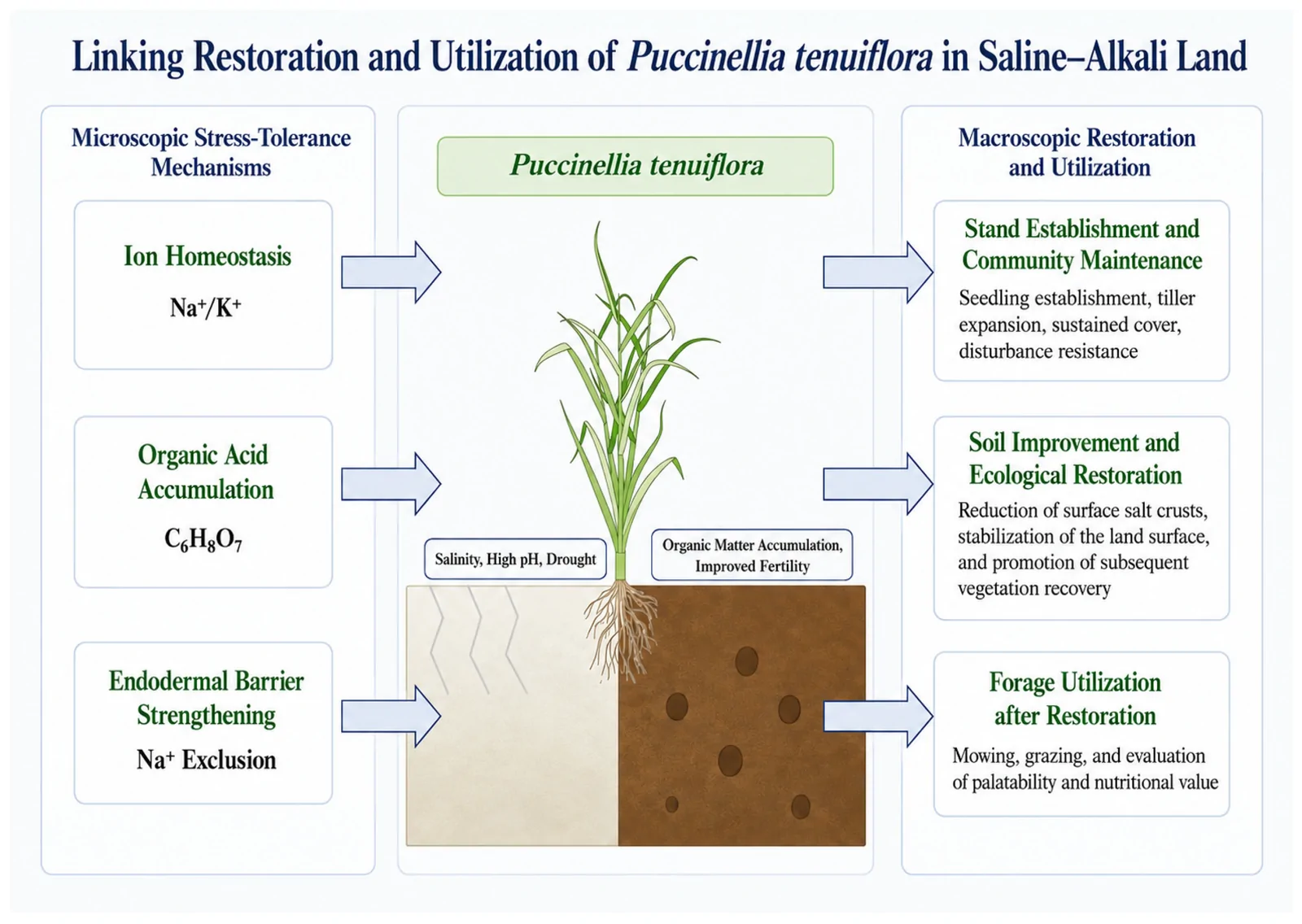
Conceptual framework of the “restoration-utilization linkage” of *P. tenuiflora* on saline–alkali land. The left side summarizes the micro-level stress tolerance basis of *P. tenuiflora*, including ion homeostasis, organic-acid metabolism, and strengthening of root barriers. The middle panel represents plant growth under combined salinity, high pH, drought, and other stresses. The right side shows community establishment, soil improvement, ecological restoration, and post-restoration forage utilization potential. The main point of the figure is straightforward: *P. tenuiflora* should first be considered a restoration plant, and forage utilization should be discussed at the post-restoration stage.

Research on *P. tenuiflora* now extends beyond description of where it grows or whether its seeds can germinate. Studies have gradually expanded to structural adaptation, ion transport, osmotic adjustment, antioxidant defense, functional gene characterization, and multi-omics responses [21,22,23,24,25,26,27,28,29,30,31,32,33,34,35,36,37,38,39,40,41,42,43,44,45,46,47,48,49,50,51,52,53]. Field establishment, community maintenance, and post-restoration utilization studies also show that the value of *P. tenuiflora* cannot be summarized simply as “salt-alkali tolerance” [10,11,12,13,14,15,16,17,18,19,20,54,55,56]. This species can translate cellular and tissue-level adaptation into community establishment on saline–alkali land, and after community stabilization it can show a certain utilization potential [20]. Therefore, this review discusses *P. tenuiflora* as part of a continuous process: stress tolerance mechanisms first, ecological restoration second, and post-restoration utilization third. Figure 1 summarizes this relationship.

## 2. Ecological Characteristics of *P. tenuiflora* and Its Restoration Significance as a Pioneer Species on Saline–Alkali Land

*P. tenuiflora* is commonly found in northern Chinese saline meadows, alkaline lowlands, and abandoned saline–alkali fields [10,11,12,13,14]. These habitats often have high salinity, high pH, poor soil structure, and unstable water and available nutrient conditions [1,2,3,4,5,7]. Many mesophytic or xerophytic crops and forages cannot establish stands easily in these sites and are even less likely to persist over time [4,7,8]. The ability of *P. tenuiflora* to survive, regreen, and gradually expand its population indicates that it can serve as an early-establishing species in degraded saline–alkali land and provide a basis for subsequent vegetation restoration [10,11,12,13,14].

Even with patchy first-year establishment and low initial density, subsequent tillering (2- to 5-fold increase) and regrowth after disturbance can substantially boost population density, reflecting a resilience mechanism driven by clonal integration and bud banks that enable rapid gap-filling and recovery from early setbacks. This pioneer role thus extends beyond initial colonization to long-term (a minimum of three consecutive growing seasons) community stabilization and gradual expansion—empirically, cover can rise from 20–30% in year one to 80–90% by year three—emphasizing that resilience, rather than first-year success, is the decisive factor in restoration outcomes [15,16,17]. Agronomic observations also indicate that this species has practical application value in alkaline grassland planting and can promote vegetation restoration [18]. Even when first-year establishment is uneven, population density can be improved gradually through tillering and regrowth. Therefore, its pioneer role is not limited to early colonization; it also includes community consolidation and continued expansion after establishment.

Comparative studies also support the application potential of *P. tenuiflora* [10,11,12,13]. In these studies, *P. tenuiflora* was evaluated alongside several representative salt-tolerant grass species (e.g., *Puccinellia distans*, *Festuca arundinacea*, and *Leymus chinensis*) under replicated field or greenhouse trials with controlled salinity gradients, and key traits such as survival rate, aboveground biomass, crude protein content, and Na^+^/K^+^ ratio were measured. Relative to other salt-tolerant grasses, *P. tenuiflora* generally shows stronger salt tolerance, better yield, and certain forage-related advantages under inland saline–alkali conditions [14]. Planting this species may also improve soil conditions in saline–alkali grasslands and promote subsequent vegetation recovery. Thus, *P. tenuiflora* should first be regarded as a pioneer grass that performs an ecological restoration function. Subsequent land use and forage should build on this restoration capacity.

Community-level studies support this interpretation [11,12,13,15,16,17,18]. Investigations of aboveground production structure, seasonal dynamics, and net primary productivity indicate that *P. tenuiflora* is a relatively stable and productive component of saline–alkali grasslands [10,11]. Studies on clonal and tiller traits further show that its population structure varies with saline–alkali habitats and disturbance [11,12,13,14,15,16,17,18,54]. Grazing and mowing can alter tiller composition and overwintering modules, indicating that persistence is closely related to management [18]. Associated plant species affect local performance through either competition or facilitation, with the net effect shifting along salinity and disturbance gradients, for example, *Suaeda salsa* may suppress Phragmites but can also facilitate later colonists by reducing rhizosphere salinity. Recognizing this context-dependency is essential for managing restoration and forage systems on saline–alkali lands [54]. Taken together, these findings indicate that *P. tenuiflora* is not a short-term colonizer that completes its role after planting. It is a pioneer species that can function over the long term in degraded saline–alkali habitats.

## 3. Structural, Physiological, and Molecular Adaptation of *P. tenuiflora* to Abiotic Stress

### 3.1. Germination and Early Seedling Responses

*P. tenuiflora* shows stress tolerance at germination and early seedling establishment, yet its germination remains sensitive to extreme pH and alkaline–salt stress [57]. In a single-factor pH experiment, seeds did not germinate under pH 2.0. At pH 3.0–5.0, germination percentage, potential, index, and vigor index were all below control levels and decreased with declining pH [57]. These results indicate that *P. tenuiflora* is not insensitive to acid–base variation, and extreme pH can still limit early establishment.

In saline–alkali land, Na_2_CO_3_ stress is closer to the practical field problem and imposes stronger inhibition on germination and early metabolism [23,58,59]. Studies show that, as alkaline–salt stress intensifies, the accumulation of soluble proteins and free amino acids during seed germination is inhibited, whereas protective solutes such as proline can remain at relatively high levels during the later stage [23,58,59]. This suggests that *P. tenuiflora* does not increase all nitrogenous metabolites; instead, it preferentially retains compounds related to osmotic protection. Threshold effects also occur at the seedling stage. Under low salt, membrane systems and photosynthetic function can be maintained, whereas stronger stress markedly increases membrane damage and photosynthetic inhibition [23,30,32,47,48,49]. Therefore, the key feature of early stress tolerance is not the complete avoidance of injury, but the maintenance of germination and establishment within a certain stress range.

### 3.2. Structural Adaptation

Structural adaptation is an important basis for *P. tenuiflora* to resist stress-induced injury. Root anatomical traits are a typical feature in studies of salt tolerance in this species. Peng et al. compared *P. tenuiflora* with wheat and found that *P. tenuiflora* roots have a more effective endodermal barrier, which can restrict Na^+^ movement toward the stele and xylem [27]. This barrier restricts apoplastic Na^+^ influx into the stele, thereby reducing Na^+^ accumulation in shoots, while higher K^+^ retention in roots maintains a favorable cytosolic K^+^/Na^+^ ratio; together, these two mechanisms enhance overall ion selectivity under saline–alkali stress. Wang et al. also suggested that low Na^+^ accumulation and a high K^+^/Na^+^ ratio are associated with restricted unidirectional Na^+^ influx in roots [28]. Thus, root structure is not merely a background condition in salt tolerance; it directly affects ion entry and long-distance transport.

Leaves and stems also undergo adaptive structural changes [29,30]. Studies of vegetative organ structure indicate that salt stress promotes strengthening of the epidermis, mechanical tissues, and vascular tissues in *P. tenuiflora* [29], which helps maintain tissue integrity and conductive continuity. Ultrastructural observations further reveal that mesophyll cells remain relatively intact under mild or moderate Na_2_CO_3_ stress, whereas progressive damage, including chloroplast swelling, thylakoid disorganization, and membrane disruption, occurs as stress intensifies [30]. These results suggest that *P. tenuiflora* cannot completely avoid structural damage, but it can delay severe injury until higher stress levels are reached.

This structural plasticity is also evident at the population level. In different saline–alkali habitats, clonal modules, tiller modules, and biomass allocation patterns are not fixed [15,16,17,18]. They adjust in response to local habitat variation. Therefore, structural adaptation in *P. tenuiflora* is not limited to microscopic features such as cell wall thickening or endodermal reinforcement. It also includes population-level adjustment of modules and resource allocation. These changes allow *P. tenuiflora* to continue forming communities in spatially heterogeneous saline–alkali environments.

### 3.3. Ion Homeostasis and Na^+^/K^+^ Selectivity

Na^+^/K^+^ homeostasis is one of the best-supported components of salt-tolerance physiology in *P. tenuiflora*. Using ^22^Na^+^ flux analysis, Wang et al. found that under 100 and 150 mM NaCl treatments, unidirectional Na^+^ influx in *P. tenuiflora* roots was approximately 37% and 31% lower than that in wheat, respectively [28]. At the same time, foliar salt loss accounted for only 0.0006% of the total plant Na^+^ [28]. This indicates that low Na^+^ accumulation in *P. tenuiflora* depends mainly on restricting Na^+^ entry through roots rather than on large-scale salt excretion from leaves. Therefore, low Na^+^ accumulation is an important phenotypic basis for understanding its salt-tolerance mechanism.

Restricted Na^+^ entry is further reflected in a higher tissue K^+^/Na^+^ ratio [27,28,35]. After 7 d of treatment with 100 mM NaCl, the shoot K^+^/Na^+^ ratio in *P. tenuiflora* remained 1.09, whereas that in wheat was only 0.24 [28]. Under higher salt concentrations, the shoot K^+^/Na^+^ ratio of *P. tenuiflora* also remained higher overall than that of wheat, indicating strong K^+^ retention and Na^+^ exclusion capacity [28]. Peng et al. also reported that *P. tenuiflora* maintains higher K^+^ and lower Na^+^ at the root tissue level and related this trait to the root endodermal barrier [27,37]. These findings suggest that salt tolerance in *P. tenuiflora* depends both on absorbing less Na^+^ and on retaining K^+^ and maintaining ion selectivity during transport.

Salt stress and alkali stress should not be treated as equivalent. Neutral salt stress mainly causes osmotic stress and ion toxicity. Alkali stress also imposes high-pH injury and is therefore more likely to cause ion imbalance and metabolic disturbance [21,22,23,24,25,60]. Taken together, these findings confirm that *P. tenuiflora* can grow normally in saline–alkali soils with pH 9–10, underscoring its exceptional tolerance to extreme alkalinity [61]. However, this does not mean that regulation under such conditions is simple. Under alkali stress, ion balance, osmotic adjustment, and root responses often participate simultaneously [21,22,24,26]. Therefore, with its contrasting responses to salt versus alkali stress, *P. tenuiflora* serves as an ideal model for comparative studies on salt-tolerance and alkali-tolerance mechanisms.

At the molecular level, ion homeostasis in *P. tenuiflora* is associated with multiple transporter systems [34,35,36]. PutHKT2;1 has characteristics related to high-affinity K^+^/Na^+^ transport and can influence the K^+^/Na^+^ relationship [34,35]. PutAKT1 is associated with K^+^ uptake and K^+^ retention under salt stress [35]. PtSOS1/PutSOS1 is localized to the plasma membrane and participates in Na^+^ efflux and ion redistribution through Na^+^/H^+^ antiport activity [35,36]. Therefore, the low Na^+^ accumulation and high K^+^/Na^+^ ratio in *P. tenuiflora* are achieved through the combined action of root structural barriers and membrane transport proteins that regulate ion uptake, efflux, and long-distance transport.

### 3.4. Osmotic Adjustment, Nitrogen Metabolism, and Organic-Acid Accumulation

Osmotic adjustment is an important part of the stress response in *P. tenuiflora*, but it is not achieved by accumulation of a single compound [21,22,23,24,25]. Under saline–alkali stress, *P. tenuiflora* accumulates compatible solutes such as proline and soluble sugars to maintain cell turgor and protect proteins and membrane systems [21,22,23,24]. Seed germination studies also show that Na_2_CO_3_ inhibits the normal accumulation of soluble proteins and free amino acids, whereas proline can still increase markedly during the later stress stage [58,59]. Therefore, osmotic adjustment in *P. tenuiflora* does not simply depend on an increase in total amino acids; rather, it preferentially accumulates specific protective solutes.

Changes in nitrogen metabolism are also stress-dependent. Strong alkaline–salt stress can suppress general nitrogen metabolism and mobilization of storage substances, while preserving key metabolic processes related to osmotic protection [23,58,59]. This selective adjustment indicates that *P. tenuiflora* does not broadly enhance all metabolic activities during early stress responses. Instead, it prioritizes processes related to cellular water balance, membrane stability, and stress buffering.

Under alkali stress, one of the most representative metabolic features of *P. tenuiflora* is the marked accumulation of organic acids, especially citric acid. Under NaCl treatment, total organic acids showed relatively little change, whereas Na_2_CO_3_ treatment induced a substantial increase, particularly in citric acid—highlighting a key physiological distinction between salt and alkali stress responses [25]. This difference suggests that the challenge caused by high pH is not limited to ion toxicity. It also involves intracellular pH buffering, charge balance, and cation–anion balance. Therefore, alkali-tolerance mechanisms in *P. tenuiflora* depend more strongly on metabolic adjustment than salt-tolerance mechanisms do.

Root secretion also participates in alkali tolerance [26]. Under alkali stress, root secretion patterns in *P. tenuiflora* change, and the secretion of phenolic acids, fatty acids, and some organic acids increases; consequently, rhizosphere regulation becomes important [26]. Studies of combined saline–alkali stress also show that roots are not only portals for ion entry but also key sites for organic-acid secretion, pH regulation, and osmotic compensation [22,24,25,26]. Thus, alkali tolerance in *P. tenuiflora* should not be understood simply as stronger salt tolerance. It involves coordinated regulation of ion homeostasis, nitrogen metabolism, and rhizosphere metabolism.

### 3.5. Antioxidant Defense, Membrane Stability, and Photosynthetic Adaptation

Salt and alkali stresses both induce oxidative injury in *P. tenuiflora*, making antioxidant defense an important component of its stress tolerance [23,32,46,47,48,49,50,51,52,53,62,63]. Seedling studies under salt stress show that at low salt levels, *P. tenuiflora* can maintain relatively low membrane lipid peroxidation through protective systems [23,32]. Once salt concentrations exceed a certain threshold, damage indicators such as malondialdehyde (MDA) content and electrolyte leakage increase markedly, indicating stronger disturbance of membrane-system stability [32,46,47,48,49,50,51,52,53].

Chlorophyll fluorescence parameters also reflect this stage-dependent pattern [30,32,49]. Under low to moderate saline–alkali stress, *P. tenuiflora* can maintain PSII reaction center activity to a certain extent and dissipate excess energy through non-photochemical quenching, state transitions, and cyclic electron transport [49]. Under severe stress, photosynthetic efficiency decreases, and structural damage to chloroplasts and vacuoles becomes more pronounced [30,32,49]. These results indicate that photosynthetic protection in *P. tenuiflora* is not a fixed state; it changes with the degree of membrane-system injury.

Gene- and protein-level studies also support this interpretation [43,44,45,46,47,48,49,50,51,52,53,64]. Genes such as PtDHAR, PtFer, PutGRXS12, and peroxisomal APX are associated with the ascorbate cycle, peroxide scavenging, and redox regulation [43,44,45,46]. Proteomic studies revealed that under salt, alkali, and low-temperature stresses, proteins involved in photosynthesis (e.g., Rubisco subunits), energy metabolism (e.g., ATP synthase), reactive oxygen species scavenging (e.g., superoxide dismutase, peroxidase), membrane stability (e.g., aquaporins), and detoxification (e.g., glutathione S-transferases) were differentially expressed, with the direction and magnitude of change varying with stress type and intensity. These findings provide molecular-level evidence for the broad adaptability of *P. tenuiflora* across multiple abiotic stresses [47,48,49,50,51,53]. Notably, a long-term salinity stress adaptation study (300 mM NaCl, two-year exposure) further revealed that under prolonged stress, *P. tenuiflora* does not simply maintain a static antioxidant response but undergoes distinct metabolic reprogramming, including enhanced accumulation of flavonoids and phenolamides for ROS scavenging, alongside specific phytohormonal adjustments (ABA upregulation, cytokinin/jasmonic acid downregulation) that sustain redox balance and tissue integrity over extended periods [65]. Therefore, antioxidant defense should not be viewed in isolation. It works together with photosynthetic regulation and energy redistribution to shape stress tolerance.

### 3.6. Molecular Transport Systems and Key Functional Genes

Several functional genes have been characterized in *P. tenuiflora*, but these genes do not act independently. A more reasonable view is to place them within interconnected processes involving membrane transport, membrane lipids and interface stability, redox buffering, and signal recognition [34,35,36,43,44,45,66,67,68,69,70]. Germin-like proteins (GLPs), as cell wall-localized proteins with dual superoxide dismutase (SOD) and oxalate oxidase (OXO) enzymatic activities, participate in reactive oxygen species (ROS) homeostasis and cell wall remodeling under stress conditions [71]. At the root level, selective ion uptake and exclusion, mediated by apoplastic barriers and membrane transporters, govern the initial entry and radial transport of Na^+^. At the shoot and organellar levels, osmotic adjustment, membrane stability, and energy metabolism then operate to cope with the Na^+^ that has entered the plant. From this perspective, molecular transport systems do more than move ions. They also influence photosynthetic maintenance, ROS control, and tissue tolerance.

**Figure 2:**
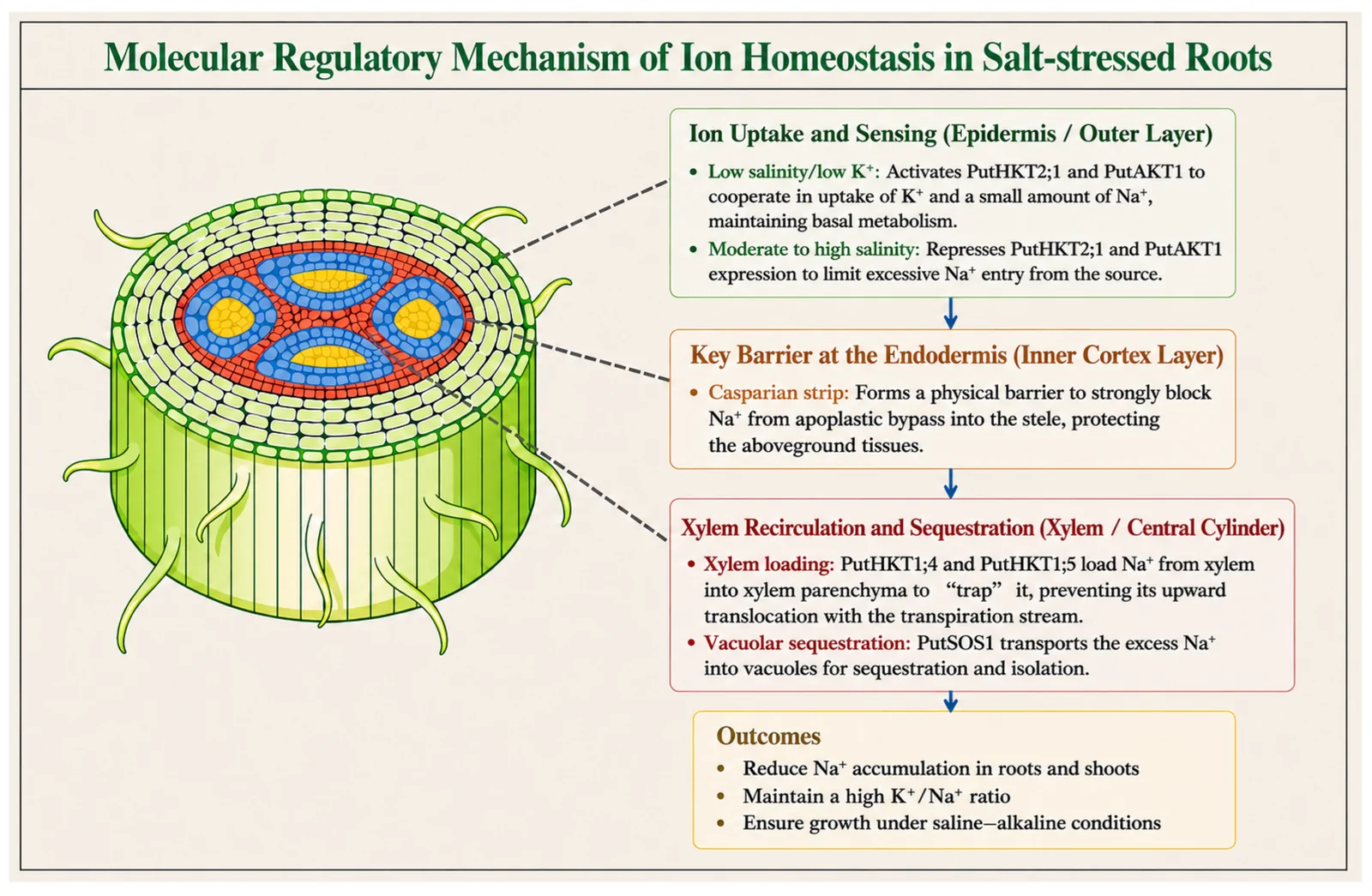
Schematic diagram of root ion homeostasis regulation in *P. tenuiflora*. The figure shows that under saline–alkali stress, *P. tenuiflora* maintains ion homeostasis through spatial coordination among the root epidermis, cortex, endodermis, and vascular tissues. The root epidermis and cortex restrict excessive Na^+^ entry and promote selective K^+^ uptake [27,28,34,35]. The endodermal barrier further suppresses Na^+^ movement toward the stele and xylem [27]. Vascular tissues participate in Na^+^ efflux, retrieval, and long-distance redistribution [28,35,36]. PutHKT2;1, PutAKT1, and PtSOS1/PutSOS1 are associated with K^+^/Na^+^ transport, selective K^+^ uptake, and Na^+^/H^+^ antiport, respectively [34,35,36]. These processes jointly maintain a high K^+^/Na^+^ ratio and thereby support saline–alkali tolerance in *P. tenuiflora*.

PutHKT2;1, PutAKT1, and PtSOS1/PutSOS1 are currently among the transporters with relatively clear mechanistic roles. PutHKT2;1 participates mainly in K^+^/Na^+^ transport and maintenance of K^+^ acquisition capacity under low-K^+^ or high-Na^+^ conditions [34,35]. PutAKT1 helps enhance selective K^+^ uptake across the plasma membrane [35]. PtSOS1/PutSOS1 contributes to Na^+^ efflux and long-distance ion redistribution through Na^+^/H^+^ antiport [35,36]. These findings indicate that *P. tenuiflora* does not survive by simply tolerating high Na^+^. Instead, it restricts Na^+^ entry, retains K^+^, and excludes or transfers the Na^+^ that has already entered. The low Na^+^, high K^+^, and elevated K^+^/Na^+^ ratio described above are plant-level outcomes of this transport relationship. The root epidermis, cortex, endodermis, and vascular tissues work together to limit Na^+^ entry, selectively absorb K^+^, and redistribute Na^+^. The major processes are shown in Figure 2.

In addition to ion transport, membrane-lipid remodeling and stress-perception-related genes may form an important interface regulatory module. PutACBP1 and its family members suggest that *P. tenuiflora* has regulatory potential in acyl-CoA binding, membrane-lipid turnover, and membrane-system stability [68,72]. PtLIR1, LLG1, EPF-type epidermal patterning peptides, LOX-related genes, and LysM receptor-related factors extend the research focus to light responses, receptor co-factors, epidermal pattern formation, extracellular signal recognition, oxylipin signaling, and pattern-recognition receptor pathways [64,69,73,74,75,76]. At present, these studies mainly provide candidate genes and expression-based functional clues. It is therefore more appropriate to state that these genes may participate, rather than to describe them as a complete causal network. High salt and high pH often first affect the plasma membrane, the cell wall–plasma membrane interface, and the extracellular ionic microenvironment. The stability of this interface may determine whether transporters function efficiently, whether ROS accumulates rapidly, and whether signals are amplified correctly.

Redox buffering is another major process that should be considered together with ion and membrane regulation. Genes such as PtDHAR, PtFer, PutGRXS12, and peroxisomal APX all point to the ascorbate-glutathione cycle, iron homeostasis, and ROS scavenging networks [43,44,45,46]. PtDHAR is related to ascorbate regeneration [43]. PtFer is related to restriction of free iron and suppression of the Fenton reaction [44]. PutGRXS12 participates in the regulation of intracellular redox status by catalyzing thiol, disulfide exchange reactions and maintaining reduced glutathione pools. This activity is critical for protecting cellular proteins from oxidative damage under abiotic stresses, as well as for modulating stress-related signaling pathways. Its involvement in redox homeostasis thus contributes to the overall stress tolerance of *P. tenuiflora* under saline–alkali conditions [45]. PutAPX is associated with peroxide scavenging and reduced H_2_O_2_ accumulation [46]. These results indicate that *P. tenuiflora* cannot resist salt stress only by using ion transporters to block salt. High Na^+^, high pH, and osmotic imbalance eventually affect membrane peroxidation and electron transport. Only when ion transport, membrane-interface stability, and redox buffering are maintained can cells keep ROS within a range that allows signaling without causing excessive damage. For this reason, tissue culture, genetic transformation, and heterologous expression platforms are important because they help determine whether candidate genes are merely correlated with stress responses or have causal functions.

### 3.7. Proteomics, Phosphoproteomics, and Multi-Omics Integration

If Section 3.6 addresses the key molecular components of *P. tenuiflora*, then proteomic, phosphoproteomic, and other omics studies address how these components are mobilized under stress. The value of multi-omics is not only that it provides more evidence. It can also move the interpretation of saline–alkali tolerance from a list of genes to a dynamic execution process. Transcriptomic and microarray studies indicate that saline–alkali stress affects metabolism, signal transduction, transcriptional regulation, and defense responses [37,38,39,40,41]. Proteomics further shows that the actual functional response is not necessarily represented by all differentially transcribed genes, but by protein modules whose abundance or post-translational modifications change in specific organs, time windows, and subcellular compartments [47,48,49,50,51,52,53].

Leaf and root proteomes first reveal clear organ-specific division of labor [47,48,49]. Under salt and alkali stress, leaves preferentially adjust photosynthesis, sugar metabolism, energy supply, membrane stability, ROS scavenging, and protein processing. The main goal is to maintain the basic operation of light energy absorption, electron transport, carbon assimilation, and detoxification [47,49]. In contrast, root proteomes emphasize Ca^2+^ signaling, restriction of Na^+^ influx, Na^+^ compartmentalization, H^+^ transport, betaine accumulation, vesicle trafficking, and protein turnover [48]. This indicates that roots are more involved in perception, screening, and redistribution, whereas leaves adjust photochemical and antioxidant resources under ion and energy pressure.

Callus proteomes further indicate that saline–alkali tolerance mechanisms are dependent on cell state. Unlike differentiated organs, in which photosynthetic maintenance is important, callus tissues emphasize ROS scavenging, glycolytic energy supply, protein folding, translation elongation, and cytoskeletal remodeling [53]. This suggests that *P. tenuiflora* does not have a single fixed stress-tolerance program that applies to all tissues. Differentiated tissues rely more on root-leaf material transport and organelle coordination. Undifferentiated tissues rely more on intracellular metabolic rearrangement, heat-shock protein chaperone systems, and antioxidant buffering to maintain survival and regeneration potential.

**Figure 3:**
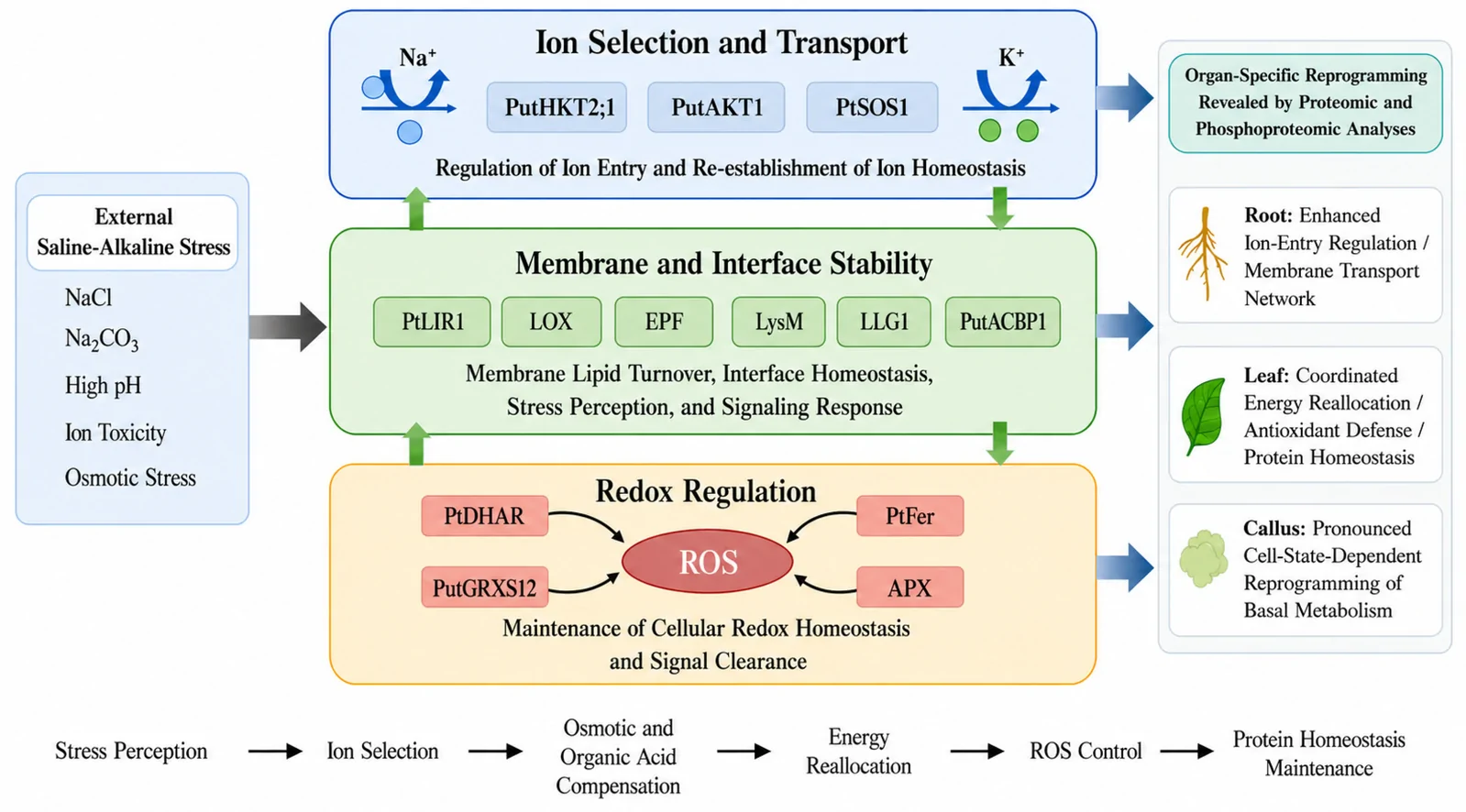
Multi-layered model of saline–alkali tolerance regulation in *P. tenuiflora*. The figure summarizes the major responses of *P. tenuiflora* to NaCl, Na_2_CO_3_, high pH, ion toxicity, and osmotic stress. Transporters such as PutHKT2;1, PutAKT1, and PtSOS1/PutSOS1 participate in restricted Na^+^ entry, K^+^ retention, Na^+^ efflux, and long-distance redistribution, which helps maintain high K^+^/Na^+^ selectivity [34,35,36]. PutACBP1, PtLIR1, LOX, LLG1, EPF, and LysM-related factors may be involved in membrane-lipid turnover, stabilization of the cell wall–plasma membrane interface, extracellular signal perception, and stress-response regulation [64,68,69,70,71,72,73,74,75,76]. PtDHAR, PtFer, PutGRXS12, and APX are associated with the AsA-GSH cycle, iron homeostasis, and ROS scavenging, which may reduce membrane peroxidation and photosynthetic electron-transport injury [43,44,45,46]. Proteomic and phosphoproteomic studies further suggest that roots, leaves, and callus tissues differ in their response priorities, involving ion transport, energy metabolism, photosystem regulation, protein homeostasis, and ROS balance [47,48,49,53]. Therefore, saline–alkali tolerance in *P. tenuiflora* is better understood as the result of multiple interacting levels rather than the effect of a single gene or pathway.

Chloroplast proteomics and phosphoproteomics shift the focus to faster regulatory processes [49]. Studies show that response proteins and phosphorylated proteins under Na_2_CO_3_ stress are mainly enriched in photosystems, state transitions, cyclic electron transport, PSII repair, ATP synthesis, Na^+^/H^+^ transport, signal transduction, and ROS homeostasis [49]. This indicates that under high-pH and high-Na^+^ conditions, *P. tenuiflora* does not simply reduce photosynthesis. Instead, it reorganizes light-energy allocation and electron-flow direction. From this perspective, saline–alkali tolerance in *P. tenuiflora* can be understood as a layered process. The outer layer first deals with ion selection and transport. The middle layer stabilizes membranes and interfaces. The inner layer maintains redox homeostasis and ROS balance. Proteomic and phosphoproteomic data reveal faster adjustments across different organs. Saline–alkali tolerance in *P. tenuiflora* is not a single-gene trait but the result of coordination among multiple processes in different tissues. Figure 3 summarizes this relationship.

## 4. Community Establishment, Post-Restoration Utilization, and Management Significance

This restoration capacity not only enables initial vegetation establishment but also determines whether the community can withstand subsequent management practices, such as rotational grazing, mowing for hay, or supplementary seeding, without losing cover or productivity. For example, a well-established *P. tenuiflora* stand can recover from moderate defoliation through rapid tillering and regrowth, making it suitable for integrated forage-livestock systems on saline–alkali land [10,11,12,13,14,15,16,17,18,19,20,54,55,56]. Field trials show that under appropriate land preparation, shallow sowing, and timely irrigation, *P. tenuiflora* can achieve emergence rates of 70–85% in abandoned saline–alkali fields, with seedling survival exceeding 80% under moderate-salinity conditions. The established populations then gradually increase community density 2- to 3-fold within two years through tillering and regrowth, demonstrating strong field performance and restoration potential [15,16,17,18,19]. In heavily salt-affected sites, first-year emergence is only the first step. The more important question is whether the population can pass the establishment bottleneck and form a persistent community. Long-term observations indicate that once established, *P. tenuiflora* stands can maintain >70% cover and stable tiller density over three to five years under moderate grazing or mowing, with clonal growth enabling gradual expansion even when first-year survival is low (e.g., <50%). These data confirm that the species can overcome the initial bottleneck and sustain community persistence beyond the first year.

The strong turf-forming ability of *P. tenuiflora* is attributed to its clonal growth strategy and flexible tillering system. Unlike many bunchgrasses (e.g., *Festuca arundinacea*) that rely solely on seedling recruitment for population maintenance, *P. tenuiflora* can rapidly fill gaps through vegetative spread via rhizomes and tillers, enabling 2- to 3-fold density increases within two years. Moreover, clonal integration—the translocation of water, nutrients, and carbohydrates between connected ramets—allows young ramets to establish under stressful conditions where seedling recruitment would fail. This strategy gives *P. tenuiflora* a distinct advantage over seed-dependent grasses in saline–alkali habitats, where germination and early seedling survival are often severely constrained by high salinity and pH. Studies of clonal modules, tiller clusters, reproductive tillers, post-fruiting vegetative tillers, and biomass allocation show that population structure varies with saline–alkali habitats [11,12,13,14,15,16,17,18]. In other words, persistence is not only static survival; it depends on dynamic adjustment of population structure. *P. tenuiflora* can buffer local environmental heterogeneity and maintain community continuity through growth allocation among different tiller types and clonal modules. For example, under mild stress, reproductive tillers dominate to ensure seed production; under moderate salinity, vegetative tillers increase to enhance clonal spread and gap-filling; and under severe stress, the plant allocates more resources to belowground bud banks and rhizome storage, reducing aboveground tiller density while maintaining long-term (a minimum of three consecutive growing seasons) population resilience.

This population-level flexibility has practical implications for field management. Once a saline–alkali grassland has been initially restored, it immediately faces questions of persistence, disturbance resistance, and subsequent use. For field managers, several practical guidelines emerge: (1) to ensure persistence, monitor cover and tiller density annually and allow at least two full growing seasons before introducing any disturbance; (2) to enhance disturbance resistance, implement rotational grazing or mowing with a 30–40 cm stubble height and a 40-day recovery interval between events; and (3) to support subsequent forage use, time the first hay harvest after seed set to balance current yield with next-season regrowth, and avoid more than two harvests per year to prevent depletion of belowground bud banks [18]. Once a saline–alkali grassland has been initially restored, it immediately faces questions of persistence, disturbance resistance, and subsequent use. In practical terms, persistence can be measured by the ability to maintain stable productivity over time—for example, sustaining annual aboveground biomass within 80–120% of the long-term average, retaining >70% cover across growing seasons, and limiting year-to-year yield fluctuation to less than 25%. These measurable outcomes provide clear benchmarks for assessing whether a restored grassland has truly passed the establishment bottleneck and achieved long-term persistence [18]. Mowing and grazing can alter tussock traits, tiller composition, and overwintering structures. To ensure sustainable use, these disturbances should be kept within recommended thresholds: for mowing, a frequency of 1–2 cuts per year with a stubble height of 5–10 cm is generally safe for *P. tenuiflora*; for grazing, a moderate stocking rate of 2–3 sheep/ha (or equivalent) and rotational systems with 30-day recovery periods between grazing events are recommended. Exceeding these thresholds—for example, mowing below 5 cm or more than three cuts per season—can reduce tiller density and impair overwintering bud banks, leading to gradual stand decline [18]. This indicates that the response of *P. tenuiflora* to management is not limited to changes in plant height or biomass. It also includes restructuring the community. Therefore, how to mow or graze after restoration, and at what intensity, will influence whether community density and productivity can be maintained over the long term.

After community establishment, the ecological significance of *P. tenuiflora* is also reflected in its contribution to community stability [11,12,13]. Studies on aboveground production structure and seasonal dynamics show that this species can persist as a relatively stable and productive component of saline–alkali grasslands [11,12,13]. Restoration plant evaluation should go beyond germination and survival to include persistence, cover formation, and ecological function. Compared with *Puccinellia distans* and *Festuca arundinacea*, *P. tenuiflora* shows superior long-term (a minimum of three consecutive growing seasons) performance: it maintains higher cover (>75%) through the third year and better survival (>80%) under high pH, whereas the others decline sharply within 2–3 years [11,12,13]. From this perspective, *P. tenuiflora* can be considered a structural species, one that provides physical framework and habitat modulation to support community stability. Unlike pioneer or dominant species, it actively shapes its environment through turf formation and soil stabilization, maintaining community integrity even under moderate disturbance, making it a foundational element in saline–alkali grassland restoration.

The continuous ecological function after restoration further increases the application value of *P. tenuiflora*. Once a community is formed, *P. tenuiflora* can stabilize the soil surface, reduce bare soil exposure, and lower the risk of secondary degradation in saline–alkali land [10,11,12,13,14]. This is especially critical in fragile inland saline–alkali regions where vegetation requires long-term maintenance. Case studies confirm this: on the Songnen Plain, two long-term restoration models achieved >90% cover and 9.5–10.5 t ha^−1^ biomass within 4–8 years; on the Inner Mongolian Plateau, vegetation recovered progressively from bare land to wetland zones; and in the Hexi Corridor, integrated measures reduced soil salinity by 1–2% and raised yields by 15%. These cases demonstrate that restored vegetation can sustain ecological function and productivity over time in these vulnerable systems. Maintaining established *P. tenuiflora* communities can reduce repeated restoration costs, improve surface cover, promote soil environmental recovery, and provide a basis for subsequent vegetation succession. Therefore, the ecological role of *P. tenuiflora* is mainly reflected in pioneer establishment, community stability, and sustained restoration of degraded saline–alkali land.

Soil amelioration is also part of the restoration value of *P. tenuiflora*. Studies have shown that its establishment can reduce soil pH by 0.5–1.0 units, decrease electrical conductivity (EC) by 20–40%, and increase soil organic matter content by 15–30% within 3–5 years of restoration, primarily through litter incorporation, root exudation, and enhanced microbial activity [11,12,13,14,77]. Existing observations indicate that planting *P. tenuiflora* can improve soil conditions in saline–alkali grasslands and contribute to subsequent vegetation development. Although the long-term mechanisms require further study, these results already show that the role of this species is not limited to aboveground cover. *P. tenuiflora* improves soil via root activity, litter return, and rhizosphere processes. Its rhizosphere harbors diverse AM fungi (40 species, 14 genera) with colonization rates of 81–86%, enhancing N/P/K uptake and reducing electrolyte leakage and proline under stress. Bacillus subtilis GB03 promotes salt tolerance by regulating Na^+^ transporter genes (*PtHKT1;5*, *PtSOS1*, *PtHKT2;1*), while soil enzyme activities (phosphatase, protease, urease) shift following its establishment. These microbial interactions collectively boost nutrient cycling, soil structure, and stress tolerance [26].

*P. tenuiflora* performance is shaped by interspecific interactions. Companion species, Leymus chinensis (competitor, gradually replaces *P. tenuiflora*), *Suaeda salsa* (facilitator, reduces salinity), and *Puccinellia distans*, exert contrasting effects on its post-restoration performance, while beneficial rhizobacteria and AM fungi enhance nutrient uptake and stress tolerance. These interactions collectively influence long-term (a minimum of three consecutive growing seasons) community dynamics. Community studies show that associated plant species can influence the local performance of *P. tenuiflora* populations [54]. Inoculation trials confirm that microbial interactions enhance stress tolerance and establishment quality in *P. tenuiflora*. AM fungi (*Funneliformis mosseae*, *Rhizophagus intraradices*) colonize roots at 81–86%, reducing conductivity and proline while improving N/P/K uptake under saline–alkali stress. Bacillus subtilis GB03 promotes growth, reduces Na^+^ accumulation, and upregulates Na^+^ transporter genes (*PtHKT1;5*, *PtSOS1*) to enhance K^+^/Na^+^ selectivity. These findings confirm that both AM fungi and beneficial rhizobacteria substantially improve early establishment under saline–alkali conditions [78,79]. These processes remain insufficiently studied under field restoration conditions, but they remind us that the long-term performance of *P. tenuiflora* depends not only on its own stress physiology but also on ecological interactions after community formation.

Overall, the significance of *P. tenuiflora* in saline–alkali ecosystems is not limited to whether it can be planted successfully. More importantly, it links stress tolerance, clonal persistence, management response, community stability, and post-restoration utilization potential [10,11,12,13,14,15,16,17,18,19,20,21,22,23,24,25,26,54,55,56,78,79]. This connection explains why *P. tenuiflora* is suitable for saline–alkali restoration. Nevertheless, limitations exist: optimal performance is confined to moderate stress (EC < 10 dS m^−1^, pH 8.5–9.5), full soil amelioration requires 3–5 years, and later-stage displacement by competitive species like Leymus chinensis may occur. Thus, *P. tenuiflora* is best deployed as part of an integrated restoration strategy rather than a standalone solution.

## 5. Post-Restoration Utilization Extension: Forage Value and Nutritional Potential of *P. tenuiflora*

*P. tenuiflora* offers usable forage on lands where conventional grasses fail. Its crude protein (8–12%) exceeds that of Leymus chinensis (6–10%) under saline conditions, with moderate fiber (NDF 55–65%, ADF 30–35%) and digestibility (55–65%) comparable to mid-quality hay and superior to Chloris virgata and *Puccinellia distans*. However, its quality is lower than alfalfa (CP 18–22%), and palatability declines after flowering [10,11,12,13,14,20,55,56]. Its unique value thus lies in providing economic returns from otherwise unproductive saline–alkali lands while delivering ecological restoration. Forage utilization should follow community stabilization, typically when aboveground dry matter reaches 2.0–2.5 t ha^−1^ (2–3 years after establishment). Sustainable use requires stubble height of 5–10 cm for mowing or residual biomass ≥1.0–1.2 t ha^−1^ after grazing; stands with annual production >3.0 t ha^−1^ support moderate use, while those below 1.5 t ha^−1^ should be rested. These thresholds balance forage production with long-term community persistence [20]. Therefore, the logic of this review should first explain why *P. tenuiflora* is suitable as a pioneer species for saline–alkali land and then discuss whether it can serve as a forage resource after restoration. This sequence better reflects the existing evidence and is closer to the practical needs of saline–alkali land management.

From an agronomic view, *P. tenuiflora* serves as a reliable forage resource due to its stable biomass production under saline–alkali conditions. Under moderate salinity, it yields 3.0–4.5 t ha^−1^—comparable to *Leymus chinensis* (2.5–4.0 t ha^−1^) and exceeding *Puccinellia distans* (1.5–2.5 t ha^−1^)—and still maintains 1.5–2.5 t ha^−1^ under high salinity. This yield stability across a wide salinity gradient, combined with moderate forage quality, makes it a dependable option where conventional grasses cannot persist [10,11,15,16,17,18,19]. Comparative studies show that this species has good fresh herbage yield and related agronomic traits in inland saline–alkali environments [10,13,14]. A relatively high leaf proportion, persistent tillering capacity, and perennial persistence also provide a morphological basis for repeated mowing or periodic grazing [11,12,13,14,15,16,17,18,19]. Importantly, stress tolerance and persistence traits should not be conflated with forage value. These traits do not by themselves indicate high forage quality or palatability; rather, they determine whether continuous harvest and further utilization are logistically possible. This distinction is particularly important in saline–alkali grasslands, because many stress-tolerant plants can survive harsh conditions but cannot necessarily provide stable and recoverable aboveground biomass for repeated use. In other words, a plant’s ability to persist does not guarantee its utility as forage; the latter depends on both sustainable yield and acceptable quality. This distinction matters because many halophytes survive but lack recoverable biomass. *Suaeda salsa* yields only 0.5–1.0 t ha^−1^ of dry matter with poor regrowth; *Chloris virgata* has limited tillering and collapses after one cut; and *Puccinellia distans* recovers slowly after defoliation. In contrast, *P. tenuiflora* combines survival with stable yields (3.0–4.5 t ha^−1^) and rapid regrowth, making it a reliable forage resource.

The available literature suggests that evaluation of the forage potential of *P. tenuiflora* should include at least several aspects [19,20,55,56,80]. The first aspect is biomass and regrowth capacity (yield, leaf–stem ratio, tiller renewal, persistence). The second is nutritional composition, with existing studies providing the following reference ranges for *P. tenuiflora* (in % of dry matter): crude protein 15.6–16.17, crude fiber 30.4–30.72, crude fat 2.3–2.52, crude ash 4.9–6.51, nitrogen-free extract 43.0–44.08, Ca and P at 0.27% and 0.47%, respectively, with elemental abundance following K > N > P > Na > Ca > Mg. These values, however, vary with growth stage and management, and should be treated as indicative rather than fixed. The third is actual animal utilization, including intake, digestibility, metabolizable energy use, and palatability [20,55,56]. The fourth is the community feedback after utilization, that is, whether mowing, grazing, or storage methods affect population structure and sustained productivity in the next growing season [18]. At present, evidence is relatively more abundant for the first and fourth aspects, whereas the second and third aspects remain clearly insufficient.

A complete standardized quality database for *P. tenuiflora* comparable to that for alfalfa or *Leymus chinensis* is lacking. Key missing parameters include CP, NDF, ADF, CF, EE, crude ash, NFE, and mineral elements (Ca, P, K, Na, Mg). Existing data are fragmented by growth stage and locality, with systematic studies on seasonal dynamics, genotype variation, and management effects still absent, limiting quality evaluation and standardization. Existing results suggest that the nutritional value of *P. tenuiflora* changes with developmental stage. Studies of seasonal dynamics of mineral elements in aboveground parts show that elemental composition is not fixed across developmental stages [19]. Harvest timing affects forage quality assessment. Optimal quality is achieved at early heading to flowering (peak CP, moderate fiber); delaying to seed-setting reduces quality. In practice, schedule the first cut at early heading, and if a second cut is taken, allow regrowth and complete it at least 30–40 days before winter to protect overwintering buds and ensure stand persistence. This is especially important for halophytic grasses, because Na, K, Ca, Mg, and ash concentrations in plant tissues are often more strongly influenced by saline–alkali environments. Reliable evaluation of *P. tenuiflora* forage quality must consider three linked factors: growth stage (quality peaks at early heading to flowering), saline–alkali gradient (moderate stress may improve some quality traits; severe stress reduces biomass and quality), and utilization method (mowing vs. grazing require different management strategies). Only by integrating these dimensions can quality be accurately assessed in a site-specific manner. Future studies should adopt standardized protocols to generate more robust nutritional data. Recommended guidelines include: (1) sampling at key phenological stages over ≥2 growing seasons; (2) ≥3 replicate sites per salinity level, with 5 quadrats per site and uniform 5 cm stubble; (3) consistent analytical methods (AOAC, Van Soest); and (4) concurrent measurement of soil EC/pH and weather data. Such standardization would greatly improve data comparability across studies and regions.

The most direct evidence for animal utilization currently comes from lamb feeding trials. Related studies show that fresh *P. tenuiflora* can increase dry matter intake, metabolizable energy intake, and crude protein intake without markedly decreasing apparent digestibility [20]. The value of this result is that it does not stop at inferring forage value from yield or chemical composition. Instead, it directly indicates that this species is usable under feeding conditions. Fresh versus hay utilization also affects nutritional value expression. In feeding trials, lambs fed fresh *P. tenuiflora* had significantly higher DMI (944 vs. 837 g day^−1^), MEI (9.1 vs. 7.9 MJ day^−1^), and CPI (86 vs. 75 g day^−1^) than those fed dried grass (*p* < 0.05), as drying caused DM loss and reduced protein availability, though digestibility did not differ. Thus, choosing between fresh grazing and hay use should consider both composition and actual nutrient intake affected by processing [20]. These results provide a basis for future comparisons of silage, mixed sowing, supplementary feeding combinations, and different preservation methods.

Other halophytes remind us that forage value is not judged by biomass alone. *Atriplex* and *Salicornia* can produce high biomass, but high salinity reduces their digestibility and nutritive value; *Suaeda* species are similarly constrained by high ash and salt content. These examples confirm that comprehensive evaluation of nutritional and anti-nutritional factors, not just biomass, is essential for practical forage utilization [20,55,56]. Forage value of *P. tenuiflora* should be assessed via an integrated framework covering three dimensions: (1) agronomic—biomass stability (≥2.0–2.5 t ha^−1^ for initial use, 3.0–4.5 t ha^−1^ steady yield), regrowth, and persistence; (2) nutritional—CP (>12–15%), fiber (NDF < 65%, ADF < 35%), and minerals (Ca ~0.27%, P ~0.47%), varying with growth stage; and (3) animal performance and safety—intake, digestibility, mineral load, and anti-nutritional factors. Only by integrating these can reliable evaluation be achieved.

Nevertheless, current evidence is not sufficient to define *P. tenuiflora* as a high-quality specialized forage crop that has already been fully evaluated [19,20,55,56]. Core indicators such as crude protein, neutral detergent fiber (NDF), acid detergent fiber (ADF), in vitro digestibility, palatability, and long-term weight-gain effects remain limited in the public literature. Relatively high mineral ions and crude ash under halophytic conditions may also raise nutritional balance and safety issues, but threshold assessments are still insufficient [19,20,55,56,80]. Management practices also affect nutritional quality, overwintering modules, tiller composition, and next-season regrowth. Long-term data from the Songnen Plain show that mowing promotes overwintering seedlings (5254 vs. 2608 individuals m^−2^) but lowers overwintering rates (76.6% vs. 84.4% under grazing); mowing increases reproductive tiller biomass (1.30- to 1.39-fold), while grazing enhances leaf biomass (1.51-fold). These contrasting effects shape tiller composition and regrowth capacity in subsequent seasons in distinct ways [18]. Therefore, the forage value of *P. tenuiflora* should not be judged from a single feeding trial or one-season yield. Animal utilization and community sustainability must be considered together.

From an application perspective, *P. tenuiflora* is better viewed as a candidate species in the “restoration-utilization linkage” of saline–alkali land rather than as a general forage crop that can replace conventional high-yield forages. In sites with severe salinity and alkalinity, where conventional forages cannot maintain persistent stands, a species that can first cover the surface, stabilize the community, and then provide a certain amount of biomass has practical significance [7,8,10,11,12,13,14,20,55,56,81]. Therefore, it is more prudent to describe *P. tenuiflora* as having post-restoration forage utilization potential. It should not be separated from the ecological restoration context and prematurely elevated to the status of a mature high-value forage resource.

## 6. Research Limitations and Future Perspectives

Future research should shift from tolerance explanation to breeding for improved tolerance. Since tolerance in *P. tenuiflora* is polygenic, multiple approaches could be prioritized: (1) marker-assisted selection once QTLs for key traits (Na^+^ exclusion, K^+^/Na^+^, organic acids) are identified; (2) genomic selection to accelerate genetic gain; (3) ecotype-based and interspecific hybridization to combine or introduce novel tolerance alleles; and (4) CRISPR/Cas9 gene editing targeting *HKT*, *SOS*, or *NHX* families. An integrated strategy combining genomic tools, phenomics, and conventional crossing is recommended for durable, broad-spectrum tolerance [21,22,23,24,25,26,27,28,34,35,36,43,44,45,47,48,49,50,51,52,53,69,70,71,72,73,74]. Multiple mechanisms are involved in the saline–alkali tolerance of *P. tenuiflora*, which can be grouped into four functional categories: (1) ion homeostasis—restricted Na^+^ entry, K^+^ retention, and ion compartmentalization; (2) osmotic regulation—organic-acid accumulation and osmotic adjustment; (3) oxidative protection—ROS buffering; and (4) molecular reprogramming—proteomic and phosphoproteomic changes. The next step is to convert these mechanisms into indicators that can be used for breeding and germplasm evaluation. Under real saline–alkali field gradients, germination and establishment, root expansion, K^+^/Na^+^ ratio, high-pH buffering capacity, regenerative tillering, and overwintering persistence should be evaluated simultaneously. Existing genomic platforms should support marker development and targeted improvement of key genes (*PutHKT2;1*, *PutAKT1*, *PtSOS1*/*PutSOS1*). Successful examples in related grasses include genome-wide SSR markers in *Leymus chinensis* (973 loci); QTL mapping for salt tolerance in *Zoysia japonica*; SRAP and DArTseq markers in *Puccinellia distans*; SSR markers in *Sporobolus virginicus* and *Aeluropus littoralis*; and QTL-linked markers (*Nax2*, *qShl-3B*) in wheat. These cases demonstrate the feasibility of applying molecular marker systems to accelerate breeding in *P. tenuiflora* [34,35,36,43,44,45,64,70,71,72]. Candidate gene discovery must be followed by functional validation using transgenic (e.g., yeast/*Arabidopsis* expression) and CRISPR/Cas9 gene editing approaches to confirm the roles of genes like *PutHKT2;1*, *PutAKT1*, and *PtSOS1*/*PutSOS1* in tolerance, providing reliable targets for breeding and genetic improvement.

Screening materials with stronger saline–alkali tolerance should not rely only on indoor stress experiments. Controlled mechanism studies are necessary, but they must be connected with field-gradient identification and multi-year persistence validation [10,11,12,13,14,15,16,17,18,19,21,22,23,24,25,26,27,28,57,58,59,79,82]. Future work should distinguish seedling survival from perennial community persistence. Long-term data from the Songnen Plain confirm that *P. tenuiflora* maintains high overwintering rates (76.6% under mowing, 84.4% under grazing) with overwintering seedling densities of 5254 vs. 2608 individuals m^−2^ under different management regimes. These quantitative baselines support evaluating persistence beyond initial establishment in real-world restoration [10,11,12,13,14,15,16,17,18,19]. Only by evaluating stress physiology, community persistence, and restoration performance together can truly usefully core materials be screened.

For post-restoration use, the key is to identify stress-tolerant populations with genuine forage potential. Here, “high-quality forage” should be defined by (1) CP > 12% (>15% excellent), NDF < 65%, ADF < 35%; (2) DMI > 2% BW, digestibility >55%; and (3) mineral safety (especially Na^+^/K^+^ balance, K^+^/Na^+^ > 2.0). These criteria provide a framework for screening populations that combine stress tolerance with practical feeding value. Existing feeding trials show that fresh *P. tenuiflora* can increase dry matter intake, metabolizable energy intake, and crude protein intake in lambs, but these results are not enough to classify it as a high-quality forage crop [20]. Future studies should evaluate agronomic traits (aboveground biomass, leaf–stem ratio, regrowth rate), nutritional quality (CP, NDF, ADF, soluble sugars, crude ash), and mineral elements (Na, K, Ca, Mg) across different saline–alkali gradients, developmental stages, and utilization methods [19,20,55,56]. These indicators should also be analyzed together with palatability, apparent digestibility, long-term weight-gain effects, and mineral-element safety thresholds.

Overall, the goal of future applied research is to move *P. tenuiflora* from a saline–alkali-tolerant pioneer grass toward a functional grass species that is more tolerant and can also be used stably after restoration. Future breeding should target stronger stress tolerance alongside stable yield, better quality, and optimized mineral composition. Relative to benchmarks—alfalfa (CP 16–25%, NDF 40–47%, ADF 33–37%) and *Leymus chinensis* (CP 6–10%)—*P. tenuiflora* shows promising CP (15.6–16.2%) and low fiber (30.4–30.7%), though comprehensive quality databases and mineral data (Na, K, Ca, Mg) remain limited. Breeding should thus aim to stabilize and optimize these parameters to match or exceed conventional forage standards [10,11,12,13,14,15,16,17,18,19,20,55,56]. Management systems for sowing, overseeding, mowing, grazing, soil amelioration, and microbial regulation also need to be improved [4,5,8]. Only when stress-tolerance breeding is connected with forage evaluation can the “restore first, use later” strategy for *P. tenuiflora* in saline–alkali land management gain stronger practical value.

## 7. Conclusions

*P. tenuiflora* is a representative pioneer grass species for the ecological restoration of saline–alkali land. Its core value lies in its ability to establish stands on degraded saline–alkali land, maintain communities, and provide a basis for subsequent vegetation recovery. Current evidence indicates that saline–alkali tolerance in *P. tenuiflora* is associated with structural barriers, Na^+^/K^+^ homeostasis, osmotic adjustment, organic-acid metabolism, antioxidant defense, and multi-level molecular regulation. At the same time, community studies, comparative trials, and limited feeding evidence suggest that its forage potential is better regarded as a post-restoration utilization extension rather than as a primary function parallel to ecological restoration. Further evidence is still needed from long-term field management, nutritional evaluation, animal validation, and mineral safety before agricultural value can be confirmed. Ultimately, however, these efforts should converge to bridge restoration and utilization—transforming saline–alkali wastelands into multifunctional systems where *P. tenuiflora* serves as a cornerstone species for both ecological recovery and sustainable forage-livestock production.

## Data Availability

No new data were created or analyzed in this review. Data sharing is not applicable to this article.
